# Duration of untreated illness of patients with obsessive–compulsive disorder in Japan

**DOI:** 10.1111/eip.13105

**Published:** 2020-12-29

**Authors:** Yoshihiro Matsumoto, Takashi Nakamae, Yoshinari Abe, Anri Watanabe, Jin Narumoto

**Affiliations:** ^1^ Department of Psychiatry, Graduate School of Medical Science Kyoto Prefectural University of Medicine Kyoto Japan

**Keywords:** health services accessibility, mental health, mental health services, obsessive–compulsive disorder, psychiatry

## Abstract

**Aim:**

Obsessive–compulsive disorder (OCD) is a common and severe disease; however, the duration of untreated illness (DUI) of OCD is approximately 7 years, which is longer than that of other psychiatric disorders. Differences in medical environments have been reported to affect the DUI. Therefore, we surveyed the DUI of OCD in Japan and the reason for delayed treatment.

**Methods:**

The study participants were outpatients who visited the OCD specialty outpatient clinic for the first time between June 1, 2017 and May 31, 2019. Obsessive–compulsive disorder was diagnosed using the criteria in the Diagnostic and Statistical Manual of Mental Disorders, 5th Edition, and semistructured clinical interviews, which included asking the reason for the delay in seeking treatment and treatment drop‐out history.

**Results:**

Seventy‐one patients met the inclusion criteria for the study. The mean period between OCD and the first visit to the hospital was 2.8 years and the mean DUI of OCD was 4.7 years. There was a significant difference in the history of tic disorders and treatment drop out between patients with a DUI of >2 years and those with a DUI of ≤2 years. The most common reason for delaying treatment was that the patient did not consider the symptoms of OCD to be those of an illness, and the most common reason for dropping out of treatment was lack of improvement.

**Conclusions:**

This was the first study on the DUI of OCD in Japan. The DUI was relatively shorter than that found by studies in other countries. Stopping treatment lengthened the duration of the illness. Preventing the patient from dropping out of treatment could further shorten the duration of the illness.

## 1 INTRODUCTION

Obsessive–compulsive disorder (OCD) is a common disorder with a lifetime prevalence of 2.3% and a 12‐month prevalence of 1.2% (Ruscio, Stein, Chiu, & Kessler, 2010). The average age at onset is 19.5 years (Ruscio et al., 2010), and it has a high recurrence rate (Sharma, Thennarasu, & Reddy, 2014). The duration of untreated illness (DUI) is often defined as the interval between the onset of a specific psychiatric disorder and the administration of the first pharmacological treatment at standard dosages for an adequate period of time. The DUI of OCD is longer than that for other serious psychiatric diseases (Altamura, Buoli, Albano, & Dell'Osso, 2010; Dell'Osso, Camuri, Benatti, Buoli, & Altamura, 2013). Studies have reported the DUI of OCD to be approximately 7 years, with the exception of some studies on OCD in India that found a DUI of 34 months (Albert et al., 2019; Altamura et al., 2010; Dell'Osso et al., 2013; Dell'Osso, Benatti, Oldani, Spagnolin, & Altamura, 2015; Dell'Osso, Buoli, Hollander, & Altamura, 2010; Viswanath, Narayanaswamy, Cherian, Reddy, & Math, 2011). Longer DUIs can lead to brain structure alterations and have been reported to cause cortical thinning in the right hemisphere (Nakamae et al., 2012), leading to reduced responses to pharmacological treatment (Albert et al., 2019; Dell'Osso et al., 2010). A reduction in the DUI may lead to better treatment outcomes resulting in earlier improvement in quality of life (QOL). To reduce the DUI, earlier access to a psychiatrist for a patient must be facilitated and dropping out of treatment must be prevented.

Differences in the medical environment were reported to affect the DUI of patients with OCD (Fineberg et al., 2019). In Japan, patients have access to the physician of their choice, even highly specialized physicians. Japan also maintains a national health insurance system (Ii, 2008), which keeps the medical expenses of the patient relatively low. Moreover, patients who use public assistance can access hospitals without any financial burden on them (Yuda, 2018). Thus, Japanese patients should have easier access to psychiatrists. A study on the barriers to mental health care in Japan showed that financial factors are a minimal barrier (Kanehara et al., 2015). Indeed, the DUI for patients in Japan with depression has been reported to be relatively short (Oguchi et al., 2014). Therefore, we analysed the DUI of OCD and the factors that contribute to it in Japan.

Discontinuation of treatment is another reason for the increase in the DUI. One study reported that the treatment drop‐out rate for psychiatric outpatients was approximately 35% (Minamisawa, Narumoto, Yokota, & Fukui, 2016); thus, discontinuation of treatment must be prevented. However, the reasons for discontinuing OCD treatment have not been sufficiently researched. Therefore, another aim of this study was to determine these reasons.

## 2 METHODS

### 2.1 Data sources

Employing a cross‐sectional study design, we obtained data from clinical records at the University Hospital, Kyoto Prefectural University of Medicine (KPUM), which has a specialty outpatient clinic for OCD patients. Approximately 40 new patients per year present for consultation. Four psychiatrists were in charge of treatment from 2017 to 2019. This study was approved by the Ethics Committee of the KPUM and was performed according to the tenets of the Declaration of Helsinki (World Medical Association, 2013).

### 2.2 Participants

Potential study participants were new outpatients who visited the OCD specialty outpatient clinic of KPUM between June 1, 2017 and May 31, 2019. To be included in the study, a patient required an OCD diagnosis according to the criteria of the Diagnostic and Statistical Manual of Mental Disorders, 5th Edition (DSM‐5). Patients who could not complete both an interview and a questionnaire were excluded from the study.

### 2.3 Data collection

The patients were interviewed by trained psychiatrists. Information obtained during the interview included the patient's sex, age, family history of psychiatric disorders, age at onset of OCD, the timing of the first visit to the hospital to seek treatment for OCD, prescription and cognitive behavioural therapy (CBT) history for OCD treatment, presence and past psychiatric comorbidities, education level, the reason for the delay in seeking help, and the reason for discontinuing treatment.

When possible, caregivers and relatives were interviewed to obtain information about the OCD onset phase of the patient because some patients had difficulty recalling the onset (Table 1).

**TABLE 1:** Sociodemographic and clinical characteristics of patients

Variables (no. of patients: *n* = 71)	
Male, *n* (%)	27 (38.0)
Age, mean (SD)	32.1 (12.7)
Education (mean number of years) (SD)	13.8 (2.5)
Age at onset of OCS, mean (SD)	20.4 (11.9)
Age at onset of OCD, mean (SD)	23.9 (11.2)
Time until first hospital visit (years), mean (SD)	2.8 (5.1)
DUI (years), mean (SD)	4.7 (7.3)
Duration of illness (years), mean (SD)	8.3 (8.5)
Comorbidity	
ASD or AD/HD, *n* (%)	14 (19.7)
Depression, *n* (%)	28 (39.4)
First treatment provider was a psychiatrist, *n* (%)	66 (93.0)

Abbreviations: AD/HD, attention‐deficit/hyperactivity disorder; ASD, autism spectrum disorder; DUI, duration of untreated illness; OCS, obsessive–compulsive symptoms; OCD, obsessive–compulsive disorder.

OCD severity was assessed using the self‐administered Yale–Brown Obsessive Compulsive Scale (Y‐BOCS) and the self‐administered Obsessive–Compulsive Inventory (OCI). We also assessed depression using the Beck Depression Inventory (BDI‐II) (Table 2).

**TABLE 2:** Demographic and clinical variables by duration of untreated illness (DUI)

Variables	All patients (*n* = 71)	DUI ≤2 (*n* = 38)	DUI > 2 (*n* = 33)	*p* ^†^
DUI (years), mean (SD)	4.7 (7.3)	0.6 (0.5)	9.5 (8.6)	—
Male, *n* (%)	27 (38.0)	15 (39.5)	12 (36.4)	.79
Age, mean (SD)	32.1 (12.7)	28.7 (12.9)	35.8 (11.4)	<.01
Age at onset of OCD, mean (SD)	23.9 (11.2)	24.6 (12.3)	23.0 (10.0)	.86
Duration of illness, mean (SD)	8.3 (8.5)	4.2 (4.4)	13.1 (9.7)	<.01
Time until first hospital visit (years), mean (SD)	2.8 (5.0)	0.4 (0.5)	5.6 (6.4)	<.01
Comorbidity				
Depression, *n* (%)	28 (39.4)	14 (36.8)	14 (42.4)	.63
ASD or AD/HD, *n* (%)	14 (19.7)	7 (18.4)	7 (21.2)	.77
History of tic disorder, *n* (%)	13 (18.3)	2 (5.3)	11 (33.3)	<.01
History of dropping out, *n* (%)	23 (32.4)	8 (21.1)	15 (45.5)	.03
Family history of psychiatric disorders, *n* (%)	40 (56.3)	24 (63.2)	16 (48.5)	.21
Family history of OCD, *n* (%)	8 (11.3)	5 (13.2)	3 (9.1)	.59
Y‐BOCS, mean (SD)	24.4 (7.6)	24.3 (8.4)	24.6 (6.7)	.89
OCI, mean (SD)	67.5 (32.2)	65.1 (32.5)	70.2 (32.2)	.58
BDI‐II, mean (SD)	23.1 (15.1)	21.6 (15.7)	24.9 (14.5)	.28

Abbreviations: AD/HD, attention‐deficit/hyperactivity disorder; ASD, autism spectrum disorder; BDI‐II, Beck Depression Inventory; DUI, duration of untreated illness; OCD, obsessive–compulsive disorder; OCI, Obsessive–Compulsive Inventory; Y‐BOCS, Yale‐Brown Obsessive–Compulsive Scale.

^†^ *p* < .05: significant.

### 2.4 Time until the first hospital visit and DUI

The time until the first visit to the hospital was defined as the period between the onset of OCD and the first visit to the hospital (including seeing physicians) to seek treatment. DUI was defined as the period between the onset of OCD and the start of taking an adequate dosage of appropriate medication for an appropriate period or undergoing adequate CBT (American Psychiatric Association, 2007). If the patient had not been treated adequately before visiting the University Hospital, we defined the date of the initial visit to the University Hospital as the beginning of adequate treatment.

### 2.5 Reasons for delaying the first hospital visit

There is no consensus regarding the delay of the first visit to the hospital; therefore, we defined it as more than a year. We provided a list of potential reasons why the patient delayed seeking help, from which the patient could choose multiple answers and could add reasons of their own. We modified the questionnaire used by Poyraz et al. (2015) (Table 3).

**TABLE 3:** Reasons for delayed hospital visit for treatment

Factors (no. of patients: *n* = 31)	Yes *n* (%)
1. Not disturbed by OCD symptoms	12 (38.7)
2. Family support for overcoming symptoms	2 (6.5)
3. Financial factors	1 (3.2)
4. Did not think OCD symptoms were associated with an illness	20 (64.5)
5. Belief that I could manage OCD symptoms	18 (58.1)
6. Possibility of having to use medication	8 (26.7)
7. I did not want to discuss OCD symptoms with psychiatrists	5 (16.1)
8. Ashamed of symptoms and of needing help	9 (29.0)
9. Spontaneous fluctuation of symptoms	6 (19.4)
10. Feeling depressed	1 (3.2)
11. Prefer to go to other department doctors or spiritual healer	1 (3.2)
12. Afraid to have a diagnosis of mental illness	5 (16.1)
13. Thinking that symptoms are related to religion/being a sinner	0 (0)
14. Perception that treatment will be ineffective	4 (12.9)
15. Thinking that symptoms are necessary to be tidy	6 (19.4)
16. Others	2 (6.5)

Abbreviation: OCD, obsessive–compulsive disorder.

### 2.6 Dropping out of treatment

Patients were considered as having dropped out of treatment if (a) they did not undergo adequate treatment and (b) their doctors wanted them to return for treatment. Furthermore, patients who asked their primary physician for referral to another clinic and patients who had received adequate treatment for OCD before terminating their hospital visits were excluded from the study. Patients were asked in open‐ended format (multiple answers were allowed) to identify the reason(s) for dropping out of treatment (Table 4).

**TABLE 4:** Reasons for dropping out of mental health treatment

Factors (no. of patients: *n* = 23)	Yes *n* (%)
I was not getting better	5 (21.7)
Treatment providers recommended using medication	3 (13.0)
Side‐effects occurred with the prescribed drug(s)	2 (8.7)
I had bad experiences with the treatment providers	4 (17.4)
I moved	2 (8.7)
I could not continue to visit the hospital because my OCD symptoms were severe	3 (13.0)
My family or caregivers wanted me to stop treatment	2 (8.7)
I have forgotten the reasons	3 (13.0)
Others	3 (13.0)

### 2.7 Statistical analysis

Patients were divided into two groups on the basis of whether their DUI was greater than or less than 2 years. There is no consensus in the literature on the definition of a long DUI; some studies have defined it as >2 years, while others have defined it as >4 years (Dell'Osso et al., 2019). Therefore, for this study, we defined a long DUI as at least 2 years. The demographic data of the two groups were compared. Summary statistics for all participants included frequencies and proportions for categorical data and medians for continuous variables. We used the chi‐square (*χ* ^2^) test for categorical variables and the Wilcoxon rank sum test for continuous variables. For all analyses, *p* < .05 (two‐tailed) was considered statistically significant. JMP ver. 14 (SAS Institute, Cary, NC) was used for all analyses.

## 3 RESULTS

### 3.1 Demographic data

Eighty‐eight patients visited the OCD clinic between June 1, 2017 and May 31, 2019, of which 84 patients were diagnosed with OCD. Seventy‐one patients completed the questionnaire and met our criteria (27 males; age range, 13–67 years; mean age, 32.1 [SD 12.7] years). The mean age at the onset of OCD was 23.9 years (SD 11.2), the mean period until the first visit to the hospital was 2.8 years, and the mean DUI was 4.7 years. The first treatment provider was a psychiatrist for 93.0% (*n* = 66) of the patients (Table 1). The difference between the time until the first visit to the hospital and the DUI was 1.9 years.

### 3.2 Comparisons between clinical variables of the two groups

Two patient groups were defined on the basis of DUI (≤2 years or >2 years) and their clinical variables were compared (Table 2). The members of the DUI > 2 years group were significantly older than the members of the DUI ≤ 2 years group. The proportion of patients with a history of tic disorders was significantly higher in the DUI > 2 years group than in the DUI ≤2 years group. The patients in the DUI > 2 years group had a significantly higher treatment drop‐out rate than did those in the DUI ≤ 2 years group. There were no significant differences in sex, age at onset of OCD, Y‐BOCS scores, OCI scores, BDI‐II scores, and the comorbidities of depression and autism spectrum disorder (ASD) or attention‐deficit/hyperactivity disorder (AD/HD) between the groups (Table 2).

### 3.3 Potential barriers to treatment

The two most common reasons given by the patients who delayed visiting the hospital for treatment (*n* = 31) were “did not think OCD symptoms were an illness” (*n* = 20; 64.5%) and “belief that I could manage OCD symptoms” (*n* = 18; 58.1%). Only 1 patient (3.2%) listed financial reasons (Table 3).

### 3.4 Reasons for dropping out of treatment

Twenty‐three patients (32.4%) dropped out of their treatment program. Some reasons for dropping out were “I was not getting better” (*n* = 5; 21.7%), “I had bad experiences with the treatment providers” (*n* = 4; 17.4%), “I could not continue to visit the hospital because OCD symptoms were severe” (*n* = 3; 13.0%), and “treatment providers recommended using medication” (*n* = 3; 13.0%) (Table 4).

## 4 DISCUSSION

### 4.1 Comparison of our findings with those of other studies

The DUI of OCD in Japan (4.7 years) was relatively shorter than that in other countries, where the mean DUI is approximately 7 years, except for one OCD study in India that found the DUI to be 34 months (Viswanath et al., 2011). Financial factors as a barrier to seeking treatment affected only one patient (3.2%) in this study. Previous studies reported that financial factors affected approximately 50% of the patients (Marques et al., 2010; Williams, Domanico, Marques, Leblanc, & Turkheimer, 2012), while one study reported a 12.5% rate (Poyraz et al., 2015). The two most common reasons for delaying seeking treatment—OCD symptoms were not considered an illness (61.3%) and belief that the symptoms could be managed (58.1%)—were consistent with those found in previous studies. We found the difference between the time until the first visit to the hospital and the DUI was approximately 2 years, similar to that in a previous report (Albert et al., 2019).

A study by Kanehara et al. (2015) on all psychiatric diseases in Japan found that the common reasons for dropping out of treatment (*n* = 24) were “I got better,” “I did not need help anymore,” and “I was not getting better.” Because that study targeted all psychiatric diseases, the common reasons for dropping out are not the same as those found in this study: “I had bad experiences with the treatment providers,” “I could not continue to visit the hospital because OCD symptoms were severe,” and “treatment providers recommended using medication.”

### 4.2 Clinical implications

The DUI of OCD in our study was relatively shorter than that found in previous studies, likely because of the lower financial burden for visiting the hospital in Japan. Financial factors were identified as the reason for delaying the visit to the hospital in only one case (3.2%).

A history of tic disorders and the drop‐out rate were significantly higher in the DUI > 2 years group than in the DUI ≤ 2 years group. Tic‐related OCD differs from non‐tic‐related OCD in many aspects, including the presentation of more sensory phenomena, familial transmission, more comorbidities, and poorer insight (Brander et al., 2019; Ekinci & Erkan Ekinci, 2020; Gomes de Alvarenga et al., 2012). Poorer insight could be directly associated with a longer DUI among tic‐related OCD patients. As expected, the DUI increased when the patient dropped out of treatment. Therefore, reducing the drop‐out rate is important to improving the DUI.

The most common reason for delayed treatment access was the patient “did not think OCD symptoms were an illness” (*n* = 20; 64.5%) There is poorer insight about tic‐related OCD; however, only 7 of the 20 patients had tic‐related OCD. Non‐tic‐related OCD patients also responded “did not think OCD symptoms were an illness”; therefore, why patients did not associate their symptoms with OCD was not only neurobiologically based but also because of a lack of knowledge about OCD. Thus, the general population needs to be educated about OCD.

Most of the doctors that the patients saw first for treating their OCD were psychiatrists. We expected that early access to a psychiatrist would decrease the period between the first visit to the hospital and receipt of adequate treatment. However, the period was approximately 2 years. Early access to a psychiatrist does not necessarily decrease the time to treatment.

The most common reason given for dropping out of treatment was that the symptoms did not improve. This indicates that the treating doctors did not increase the dose of medication to an adequate level or provide adequate CBT, even though the OCD symptoms of their patients did not improve. This situation is likely due to the psychiatrists misidentifying OCD symptoms (Glazier, Calixte, Rothschild, & Pinto, 2013). Fear of medication (including side‐effects) and the inability to visit the hospital because of the severity of OCD symptoms were other reasons for dropping out of treatment. Providing adequate CBT could prevent patients from dropping out, but providing CBT is difficult in many countries (Brakoulias et al., 2019). Telemedicine or outreach also could prevent patients with severe OCD from dropping out of treatment.

### 4.3 Study limitations

This study had several limitations. First, the study was conducted at only one university hospital, and the number of participants was small. Therefore, our results may not be generalizable for use by other hospitals in Japan. However, the number of participants in our study was almost the same as the average number of participants in other studies on the DUI of OCD. Second, we defined the date that the patient first visited the University Hospital as the date that adequate treatment began for patients who had not previously received adequate treatment. Therefore, the DUI in this study was likely shorter than the actual DUI. However, this difference is only several months, at most. Third, this was a cross‐sectional study, and DUI was assessed retrospectively. We relied on information provided by the patients and available family members, which may have been inaccurate.

## 5 CONCLUSION

This was the first study on the DUI of OCD in Japan. It found that the DUI of OCD was relatively shorter than that reported in previous studies conducted in other countries. The lengths of the DUI of patients with a history of tic disorders and treatment drop out were significantly different. Educating the public about OCD could help shorten the DUI by decreasing the time until the patient first visits the hospital. Moreover, increasing the number of facilities that provide CBT, telemedicine, or outreach could further prevent a patient from dropping out of treatment.

## CONFLICT OF INTEREST

The authors declare no conflicts of interest.

## Data Availability

Data supporting the findings are available from the authors upon request.
